# Emergency Surgical Repair for Acute Type A Aortic Dissection in Octogenarians: A Single-Centre Experience

**DOI:** 10.7759/cureus.112123

**Published:** 2026-07-06

**Authors:** Yuh Ing Lok, Bao Nguyen, James Kuo

**Affiliations:** 1 Cardiothoracic Surgery, Derriford Hospital, Plymouth, GBR

**Keywords:** acute type a aortic dissection, emergency aortic surgery, octogenarians, surgical outcomes, survival analysis

## Abstract

Emergency surgical repair for acute type A aortic dissection (ATAAD) in octogenarians remains controversial due to the perceived high operative risk. This retrospective cohort study aimed to describe in-hospital mortality, major postoperative morbidity, and mid-term survival among 23 consecutive octogenarians who underwent emergency surgical repair for ATAAD between 2014 and 2024. In-hospital mortality occurred in 5 of 23 patients (21.7%). Postoperative stroke occurred in 6 of 23 patients (26.1%), and temporary renal replacement therapy was required in 6 of 23 patients (26.1%). Estimated five-year survival was 43% (95% CI 21-65%), with a median survival of 3.88 years. Despite advanced age and significant perioperative risk, these findings demonstrate that acceptable survival outcomes can be achieved and are comparable to those reported in the International Registry of Acute Aortic Dissection (IRAD). Age alone should not preclude surgical intervention, and decisions should be individualised based on patient comorbidities, functional status, and preferences.

## Introduction

Acute type A aortic dissection (ATAAD) is a life-threatening aortic condition requiring emergency surgical intervention, with mortality increasing rapidly within the first 48 hours without treatment [1]. Contemporary management guidelines recommend urgent surgical repair whenever feasible, as medical therapy alone is associated with poor outcomes [2,3]. Although advances in operative techniques, cerebral protection strategies, and perioperative care have improved outcomes, ATAAD continues to be associated with substantial morbidity and mortality.

As the global population continues to age, the number of elderly patients presenting with ATAAD has steadily increased [4]. Previous studies from the International Registry of Acute Aortic Dissection (IRAD) and other contemporary series have demonstrated that emergency surgical repair provides a significant survival benefit compared with medical management alone, even in octogenarians [5-8]. However, operative mortality remains higher among elderly patients than in younger cohorts [5,7,9]. Furthermore, surgical intervention is not undertaken in up to 28% of elderly patients, often because of advanced age, comorbidities, patient refusal, intramural haematoma, or death before planned surgery [5]. These findings highlight the ongoing uncertainty regarding patient selection and optimal management strategies in this high-risk population.

Contemporary real-world data from United Kingdom centres remain limited, particularly regarding mid-term survival following emergency ATAAD repair in octogenarians. In addition, the balance between the potential benefits of surgery and the increased perioperative risks associated with advanced age remains a subject of debate. Therefore, the objective of this study was to describe in-hospital mortality, major postoperative morbidity, and mid-term survival following emergency surgical repair of ATAAD in octogenarians treated at a UK tertiary cardiac centre.

## Materials and methods

Patients

This retrospective observational cohort study included 23 consecutive patients aged 80 years or older who underwent emergency surgical repair for ATAAD at our institution between January 2014 and December 2024. The diagnosis in all patients was established by contrast-enhanced computed tomography scan (CECT), and no coronary angiography was performed. Patients with subacute or chronic type A aortic dissection, as well as those with iatrogenic aortic dissection, were excluded. Clinical data were obtained from the National Adult Cardiac Surgery Audit database. Collected variables included patient demographics, preoperative clinical characteristics, operative details such as circulatory arrest time, postoperative complications, and survival outcomes. All data were anonymised, and no patient-identifiable information was accessible to the authors during or after data collection.

Operative techniques

Following arterial cannulation via the common femoral artery (n = 22) or axillary artery (n = 1), all procedures were performed through a standard median sternotomy. All patients underwent first-time sternotomy with no redo procedures. Cardiopulmonary bypass (CPB) was established using a two-stage venous cannula inserted into the right atrium.

Systemic cooling was initiated, with the degree of hypothermia determined by the operating surgeon and planned operative strategy. Six patients were initially cooled to 32°C. After aortic cross-clamping and transection of the distal ascending aorta, the location of the intimal tear and the anatomy of the aortic root and valve were assessed to determine whether aortic root reconstruction or replacement was required. Myocardial protection was achieved using intermittent cold blood cardioplegia delivered directly into the coronary ostia. The distal anastomosis technique was determined by the extent of aortic involvement. In three patients with the intimal tear confined to the ascending aorta, a closed distal anastomosis was performed under aortic cross-clamp without deep hypothermic circulatory arrest (DHCA), and the ascending aorta was replaced with a gelatin-impregnated Dacron graft (Terumo Aortic; Glasgow, Scotland, UK), with aortic valve resuspension performed during the proximal anastomosis. In the other three patients with more extensive aortic involvement, cooling was continued to 22°C, and an open distal anastomosis was performed under DHCA. The remaining 17 patients underwent DHCA with cooling directly to 22°C and an open distal anastomosis without aortic cross-clamping.

Among the 20 patients undergoing DHCA, antegrade cerebral perfusion was used in four patients, retrograde cerebral perfusion in 13 patients, and no adjunctive cerebral perfusion in three patients. In patients with extension of the intimal tear into the aortic arch, hemiarch or total arch replacement was performed. Following completion of the distal anastomosis, the graft was clamped, CPB was resumed, and the proximal anastomosis was completed with aortic valve resuspension. Cell salvage and cerebral oximetry were routinely utilised intraoperatively to optimise blood conservation and monitor cerebral perfusion.

Clinical outcomes

The primary outcome of interest was operative mortality, defined as in-hospital mortality. Secondary outcomes included mid-term survival and major postoperative complications, including new postoperative stroke, new requirement for renal replacement therapy, chest re-exploration for bleeding, low cardiac output syndrome, tracheostomy, and length of hospital stay.

Statistical analysis

Continuous variables were reported as mean ± standard deviation. Categorical variables were presented as frequencies and percentages. Survival was evaluated using Kaplan-Meier analysis, with median survival and five-year survival estimates reported with 95% confidence intervals (CIs).

Univariable Cox proportional hazards regression was performed to identify predictors of all-cause mortality during follow-up. Results were reported as hazard ratios (HRs) with 95% CI, Wald chi-square (Wald χ^2^) statistics, and corresponding p-values. Associations between pre-operative and operative factors and postoperative stroke were assessed using univariable logistic regression. Results were reported as odds ratios (ORs) with 95% CI, Wald χ^2^ statistics, and corresponding p-values.

Given the small sample size and limited number of outcome events, all regression analyses were considered exploratory and hypothesis-generating. A p-value of <0.05 was considered statistically significant.

## Results

Baseline characteristics

The cohort comprised 23 octogenarians undergoing emergency ATAAD repair, as shown in Table 1, with a mean age of 82 ± 2 years and a predominance of male patients (13 patients, 56.5%). Hypertension was the most common comorbidity, affecting 17 patients (73.9%), while coronary artery disease and chronic kidney disease were each present in five patients (21.7%). A history of transient ischaemic attack was identified in four patients (17.4%). The mean European System for Cardiac Operative Risk Evaluation II (EuroSCORE II) was 9.6 ± 7.3, reflecting the high operative risk profile of this population [10]. Most patients had preserved left ventricular ejection function (LVEF), with good LVEF (>50%) observed in 13 patients (56.5%) and moderate LVEF (31-50%) in 10 patients (43.5%).

**Table 1:** Baseline characteristics of octogenarians undergoing emergency ATAAD repair Continuous variables are presented as mean ± standard deviation (range) and categorical variables as n (%). ATAAD: acute type A aortic dissection; EuroSCORE II: European System for Cardiac Operative Risk Evaluation II

Variable	Value
Demographics	
Age, years (mean ± SD)	82 ± 2 (80-86)
Male sex, n (%)	13 (56.5)
Comorbidities, n (%)	
Hypertension	17 (73.9)
Diabetes mellitus	1 (4.3)
Smoking history	12 (52.3)
Chronic obstructive pulmonary disease (COPD)	1 (4.3)
Coronary artery disease	5 (21.7)
Cerebrovascular disease	4 (17.4)
Chronic kidney disease	5 (21.7)
Pre-operative dialysis	0 (0)
Connective tissue disease	2 (8.7)
EuroSCORE II, % (mean ± SD)	9.6 ± 7.3
Left ventricular ejection fraction, n (%)	
Good (>50%)	13 (56.5)
Moderate (31-50%)	10 (43.5)
Poor (£30%)	0 (0)
Dissection-related status, n (%)	
Aortic valve insufficiency	6 (26.1)
Cardiac tamponade	1 (4.3)
Malperfusion, n (%)	
Coronary	1 (4.3)
Brain	1 (4.3)
Renal	4 (17.4)
Mesenteric	1 (4.3)
Lower limb	0 (0)
Shock	1 (4.3)
Preoperative cardiopulmonary resuscitation	0 (0)

The preoperative dissection-related findings included varying degrees of aortic insufficiency in six patients (26.1%), while one patient (4.3%) developed cardiac tamponade. Malperfusion complications included coronary malperfusion presenting with myocardial ischaemia in one patient (4.3%), transient ischaemic attack with full neurological recovery prior to surgery in one patient (4.3%), renal malperfusion with acute kidney injury in four patients (17.4%), and mesenteric ischaemia in one patient (4.3%). One patient (4.3%) also presented in shock, requiring preoperative inotropic support.

Operative details

The operative details are summarised in Table 2. The majority of patients underwent ascending ± hemiarch replacement (20 patients, 86.9%), while three patients (13.0%) required total arch replacement with frozen elephant trunk implantation due to extension of the intimal tear beyond the proximal aortic arch. Concomitant procedures included coronary artery bypass grafting in four patients (17.4%), including one patient with coronary malperfusion secondary to dissection extension into the coronary ostium and three patients with significant coronary artery disease identified on preoperative CT imaging. Aortic valve resuspension was performed during the proximal anastomosis in all patients, and none required aortic valve replacement. Mitral valve repair was performed in one patient (4.3%) following identification of severe mitral regurgitation on intraoperative transoesophageal echocardiography. Femoral artery cannulation was the predominant cannulation strategy, utilised in 22 patients (95.7%), and DHCA was used in 20 patients (86.9%). In patients with a limited entry tear confined to the ascending aorta without extension into the aortic arch, the distal anastomosis was performed with the aortic cross-clamp in place without the use of DHCA. The mean CPB time, aortic cross-clamp time, and DHCA time were 211 ± 67 minutes, 89 ± 29 minutes, and 17 ± 11 minutes, respectively.

**Table 2:** Operative characteristics of octogenarians undergoing emergency repair for acute type A aortic dissection (ATAAD) Continuous variables are presented as mean ± standard deviation (range), and categorical variables as n (%). DHCA: deep hypothermic circulatory arrest

Variable	Value
Primary entry tear, n (%)	
Ascending aorta	18 (78.3)
Arch of aorta	5 (21.7)
Aortic root	0 (0)
Procedure performed, n (%)	
Ascending ± hemiarch replacement	20 (86.9)
Total arch replacement (frozen elephant trunk)	3 (13.0)
Aortic root replacement	0 (0)
Concomitant procedures, n (%)	
Coronary artery bypass grafting	4 (17.4)
Aortic valve replacement	0 (0)
Mitral valve repair	1 (4.3)
Cannulation site, n (%)	
Femoral artery	22 (95.7)
Axillary artery	1 (4.3)
Neuroprotection strategy, n (%)	
DHCA	20 (86.9)
Antegrade cerebral perfusion	4 (17.4)
Retrograde cerebral perfusion	13 (56.5)
No cerebral perfusion	3 (13.0)
No DHCA	3 (13.0)
Operative times, min (mean ± SD)	
Cardiopulmonary bypass time	211.1 ± 66.6 (93-323)
Cross-clamp time	89.2 ± 28.8 (47-160)
Circulatory arrest time	17.1 ± 11.3 (0-33)

Postoperative outcomes

Postoperative outcomes demonstrated new-onset stroke in six patients (26.1%), including transient neurological deficit with complete recovery in three patients, mild permanent residual neurological deficit in two patients, and fatal severe stroke in one patient, as shown in Table 3. Temporary renal replacement therapy was required in six patients (26.1%). Bleeding requiring chest reopening occurred in one patient (4.3%), while low cardiac output syndrome and tracheostomy were each observed in two patients (8.7%). The mean length of hospital stay was 18 ± 13 days. In-hospital mortality occurred in five patients (21.7%), and the causes of mortality are listed in Table 3. Among the 18 surviving patients (78.3%), five were discharged home, four were transferred to rehabilitation facilities, and nine were transferred back to their referring hospitals. At follow-up, no late aortic reinterventions were performed.

**Table 3:** Postoperative complications, length of hospital stay, and in-hospital mortality following emergency repair of acute type A aortic dissection (ATAAD) in octogenarians Continuous variables are presented as mean ± standard deviation (range), and categorical variables as n (%).

Variable	Value
Postoperative complications, n (%)	
New-onset stroke	6 (26.1)
New renal replacement therapy (temporary)	6 (26.1)
Bleeding requiring chest reopening	1 (4.3)
Low cardiac output syndrome	2 (8.7)
Tracheostomy	2 (8.7)
Length of stay	
Hospital stay, days (mean ± SD)	18 ± 13 (3-56)
Operative mortality, n (%)	
In-hospital mortality	5 (21.7)
Intractable bleeding	1 (4.3)
Right heart failure	2 (8.7)
Severe stroke	1 (4.3)
Severe sepsis secondary to pneumonia	1 (4.3)

Survival

**Figure 1:**
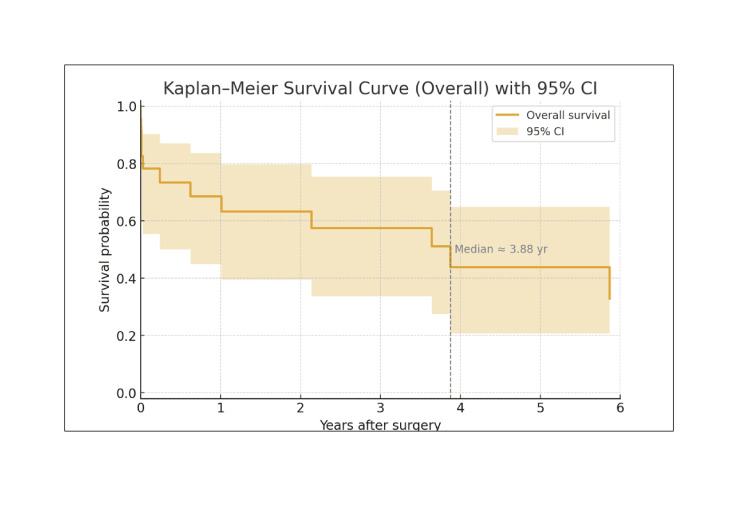
Kaplan-Meier survival curve demonstrating all-cause mortality following emergency surgical repair of acute type A aortic dissection (ATAAD) in octogenarians The estimated five-year survival was 43% (95% CI 21-65%), with a median survival of 3.88 years.

Kaplan-Meier analysis demonstrated a five-year survival rate of 43% (95% CI 21-65%) with a median survival of 3.88 years following emergency ATAAD repair in octogenarians, as shown in Figure 1.

Risk factor analysis

Risk factor analysis identified pre-operative transient ischaemic attack as significantly associated with increased mortality, as shown in Tables 4-5. Longer circulatory arrest time demonstrated a trend toward higher mortality and postoperative stroke, although this did not reach statistical significance. Frozen elephant trunk repair also demonstrated higher ORs for postoperative stroke.

**Table 4:** Univariable analysis of pre-operative and operative factors associated with in-hospital mortality following emergency repair of acute type A aortic dissection (ATAAD) in octogenarians Hazard ratios (HRs) are presented for mortality. Regression analyses were exploratory due to the small sample size and limited number of events. χ^2^: chi-square; CI: confidence interval; COPD: chronic obstructive pulmonary disease; LVEF: left ventricular ejection fraction; CPB: cardiopulmonary bypass; DHCA: deep hypothermic circulatory arrest; EuroSCORE II: European System for Cardiac Operative Risk Evaluation II

Predictor	Mortality HR (95% CI)	Wald χ^2^	P-value
Pre-operative factors			
Age	0.73 (0.37-1.45)	0.80	0.37
Male sex	1.78 (0.25-12.45)	0.34	0.56
EuroSCORE II	0.81 (0.58-1.13)	1.57	0.21
Chronic kidney disease	1.01 (0.98-1.03)	0.17	0.68
Diabetes	Not estimable	-	-
Hypertension	1.32 (0.29-5.95)	0.13	0.72
Smoking history	1.09 (0.28-4.18)	0.02	0.90
COPD	1.64 (0.19-13.9)	0.21	0.65
Cerebrovascular disease	4.40 (1.22-15.9)	5.10	0.024
Coronary artery disease	0.91 (0.24-3.42)	0.02	0.89
LVEF	1.47 (0.39-5.57)	0.32	0.57
Operative factors			
CPB time (per minute)	1.01 (0.99-1.02)	1.03	0.31
Cross-clamp time (per minute)	1.01 (0.99-1.03)	1.17	0.28
DHCA time (per minute)	1.03 (0.99-1.08)	2.87	0.09
Antegrade cerebral perfusion	2.10 (0.37-11.9)	0.71	0.40
Retrograde cerebral perfusion	0.88 (0.22-3.55)	0.03	0.86
Frozen elephant trunk	2.45 (0.42-14.3)	0.99	0.32

**Table 5:** Univariable analysis of pre-operative and operative factors associated with new-onset stroke following emergency repair of acute type A aortic dissection (ATAAD) in octogenarians Odds ratios (ORs) are presented for new-onset stroke. Regression analyses were exploratory due to the small sample size and limited number of events. χ^2^: chi-square; CI: confidence interval; COPD: chronic obstructive pulmonary disease; LVEF: left ventricular ejection fraction; CPB: cardiopulmonary bypass; DHCA: deep hypothermic circulatory arrest; EuroSCORE II: European System for Cardiac Operative Risk Evaluation II

Predictor	Stroke OR (95% CI)	Wald χ^2^	P-value
Pre-operative factors			
Age	0.89 (0.70-1.13)	0.92	0.34
Male sex	1.80 (0.24-13.5)	0.34	0.56
EuroSCORE II	0.93 (0.79-1.09)	0.84	0.36
Chronic kidney disease	1.00 (0.98-1.02)	0.01	0.92
Diabetes	Not estimable	-	-
Hypertension	1.41 (0.27-7.29)	0.16	0.69
Smoking history	1.25 (0.28-5.59)	0.09	0.77
COPD	1.82 (0.18-18.5)	0.26	0.61
Cerebrovascular disease	Not estimable	-	-
Coronary artery disease	0.88 (0.20-3.82)	0.03	0.86
LVEF	1.62 (0.37-7.01)	0.41	0.52
Operative factors			
CPB time (per minute)	1.03 (0.99-1.06)	2.55	0.11
Cross-clamp time (per minute)	1.02 (0.99-1.05)	1.80	0.18
DHCA time (per minute)	1.04 (0.99-1.09)	3.06	0.08
Antegrade cerebral perfusion	7.50 (0.70-80.4)	1.89	0.17
Retrograde cerebral perfusion	0.55 (0.11-2.77)	0.52	0.47
Frozen elephant trunk	7.50 (0.70-80.4)	1.89	0.17

## Discussion

Emergency surgical repair for ATAAD in octogenarians remains challenging because of advanced age, frailty, and multiple comorbidities. Historically, surgical intervention in elderly patients has been associated with mortality rates exceeding 40% [7], leading some clinicians to favour conservative management. However, medical treatment alone for ATAAD is also associated with an extremely poor prognosis, with mortality increasing by 1-2% per hour and reaching as high as 60-70% within the first week [11]. Contemporary international guidelines therefore continue to recommend emergency surgical repair whenever feasible [2,3].

In our cohort, carefully selected octogenarians demonstrated acceptable early and mid-term outcomes despite the high operative risk profile. The observed in-hospital mortality occurred in 5 of 23 patients (21.7%), which lies within the range of outcomes reported in the IRAD surgical mortality of 25.1% in octogenarians and remained substantially lower than the 52% in-hospital mortality observed with medical management alone [5]. Similar findings have been reported by Suzuki et al., who demonstrated a hospital mortality of 10.9% in octogenarians and found no significant differences in mortality or major morbidity compared with younger patients [12]. Park et al. likewise reported acceptable outcomes in carefully selected octogenarians, further supporting the feasibility of surgical intervention in appropriately selected elderly patients [4].

Our study demonstrated a five-year survival of 43% with a median survival of 3.88 years. These findings are in keeping with previously published IRAD data, which reported a five-year survival of approximately 35% in octogenarians undergoing surgical repair [3]. Other contemporary studies have similarly shown that meaningful long-term survival can be achieved following emergency ATAAD repair in elderly patients [8,13,14]. Given that the contemporary UK median age at death is 82.3 years for men and 85.8 years for women [15], advanced age alone should not be considered an absolute contraindication to surgical intervention.

Postoperative neurological complications and renal dysfunction remained common in this cohort, reflecting both the complexity of ATAAD surgery and the reduced physiological tolerance of elderly patients to major aortic intervention. Risk factor analysis demonstrated an association between pre-operative transient ischaemic attack and increased mortality, while longer circulatory arrest time showed a trend towards higher mortality and postoperative stroke. Frozen elephant trunk repair also demonstrated higher ORs for postoperative stroke, likely related to the greater operative complexity and prolonged extracorporeal circulation and circulatory arrest required for these procedures. These observations are consistent with a previous study, which identified prolonged operative times, extensive arch reconstruction, and advanced age as important contributors to postoperative morbidity and mortality [16]. Therefore, a conservative surgical strategy with limited aortic replacement may be favourable in selected elderly patients to reduce operative duration and postoperative complications while maintaining acceptable survival outcomes [17]. However, frozen elephant trunk repair may be unavoidable in selected patients with intimal tear extension beyond the proximal arch, where more extensive arch intervention is required to achieve adequate exclusion of the primary tear.

Limitations

This study has several limitations. It is a retrospective single-centre analysis with a relatively small sample size and a limited number of events, resulting in wide CIs and reduced statistical power. Consequently, the regression analyses should be regarded as exploratory and hypothesis-generating rather than as establishing definitive causal relationships. In addition, selection bias is unavoidable, as only patients considered suitable for surgery were included. Data on patients managed non-operatively or who declined surgery were unavailable because the study was based on a retrospective surgical database, precluding comparison with conservatively managed patients. Owing to the retrospective nature of the study, data on frailty, postoperative quality of life, and long-term neurological disability following stroke were unavailable, limiting assessment of functional outcomes. Clinical follow-up was not standardised because patients were managed by multiple surgeons. Consequently, the mean clinical follow-up duration was not assessed, and long-term clinical outcomes, including hospital readmissions to peripheral hospitals, could not be reliably evaluated. Finally, our findings are discussed in the context of the existing literature, and comparisons with previously published studies should be interpreted as providing context rather than direct comparative evidence.

## Conclusions

Emergency surgical repair for ATAAD in carefully selected octogenarians can achieve acceptable early and mid-term outcomes despite the high operative risk profile. In-hospital mortality and mid-term survival were comparable with contemporary international data, supporting the role of surgical intervention in this elderly population. Although postoperative neurological and renal complications remain common, meaningful survival can still be achieved, and advanced age alone should not be considered an absolute contraindication to emergency ATAAD repair. Operative decision-making should remain individualised, taking into consideration patient wishes, comorbidities, anatomical complexity, and premorbid functional status.
